# Development of a new fluorescent reporter:operator system: location of AraC regulated genes in *Escherichia coli* K-12

**DOI:** 10.1186/s12866-017-1079-2

**Published:** 2017-08-03

**Authors:** Laura E. Sellars, Jack A. Bryant, María-Antonia Sánchez-Romero, Eugenio Sánchez-Morán, Stephen J. W. Busby, David J. Lee

**Affiliations:** 1 Institute of Microbiology and Infection, School of Biosciences, University of Birmingham, Edgbaston, Birmingham, B15 2TT UK; 2 Departamento de Genética, Facultad de Biología, Universidad de Sevilla, 41080 Seville, Spain; 3 School of Biosciences, University of Birmingham, Edgbaston, Birmingham, B15 2TT UK; 4 Department of Life Sciences, Birmingham City University, Edgbaston, Birmingham, B15 3TN UK

**Keywords:** FROS, GFP, Fluorescent microscopy, Chromosome, Nucleoid, *Escherichia coli*

## Abstract

**Background:**

In bacteria, many transcription activator and repressor proteins regulate multiple transcription units that are often distally distributed on the bacterial genome. To investigate the subcellular location of DNA bound proteins in the folded bacterial nucleoid, fluorescent reporters have been developed which can be targeted to specific DNA operator sites. Such Fluorescent Reporter-Operator System (FROS) probes consist of a fluorescent protein fused to a DNA binding protein, which binds to an array of DNA operator sites located within the genome. Here we have developed a new FROS probe using the *Escherichia coli* MalI transcription factor, fused to mCherry fluorescent protein. We have used this in combination with a LacI repressor::GFP protein based FROS probe to assess the cellular location of commonly regulated transcription units that are distal on the *Escherichia coli* genome.

**Results:**

We developed a new DNA binding fluorescent reporter, consisting of the *Escherichia coli* MalI protein fused to the mCherry fluorescent protein. This was used in combination with a Lac repressor:green fluorescent protein fusion to examine the spatial positioning and possible co-localisation of target genes, regulated by the *Escherichia coli* AraC protein*.* We report that induction of gene expression with arabinose does not result in co-localisation of AraC-regulated transcription units. However, measurable repositioning was observed when gene expression was induced at the AraC-regulated promoter controlling expression of the *araFGH* genes, located close to the DNA replication terminus on the chromosome. Moreover, in dividing cells, arabinose-induced expression at the *araFGH* locus enhanced chromosome segregation after replication.

**Conclusion:**

Regions of the chromosome regulated by AraC do not colocalise, but transcription events can induce movement of chromosome loci in bacteria and our observations suggest a role for gene expression in chromosome segregation.

**Electronic supplementary material:**

The online version of this article (doi:10.1186/s12866-017-1079-2) contains supplementary material, which is available to authorized users.

## Background

Bacterial nucleoids are highly compacted structures composed of chromosomal DNA, nucleoid structuring proteins and RNA [1]. The DNA within the *Escherichia coli* K-12 nucleoid is folded into a structure consisting of four independently folded macrodomains, and two non-structured regions [2–4]. Each domain is located at a distinct position within the cell and the DNA within each domain appears isolated from the rest of the chromosome. Despite this, there is evidence to suggest that, at some level, the nucleoid organisation allows for spatial repositioning of active transcription units and clusters of commonly regulated genes. Qian et al. [5] exploiting a chromatin conformation capture technique, demonstrated that the *E. coli* GalR transcription repressor protein, associated with DNA target sites in different macrodomains, could co-localise. Also, a plasmid-encoded transcription unit can re-locate to particular cellular positions when being actively expressed [6].

To investigate these points, we have exploited the *E. coli* AraC regulon. AraC is a transcription activator that regulates genes involved in the uptake and metabolism of arabinose. AraC binds to its DNA target in the absence of arabinose, and activates transcription of four transcription units, located in three different macrodomains, only in the presence of arabinose [7]. Thus, in this study we have introduced Fluorescent Reporter-Operator System probes (FROS probes) [8–12], adjacent to AraC regulated promoters, to observe their cellular location and any spatial repositioning that occurs upon induction of transcription by arabinose. To facilitate this, we developed a FROS probe based on the *E. coli* MalI DNA binding protein [13, 14], fused to mCherry fluorescent protein, and its cognate DNA target site. In combination with a modified LacI:GFP FROS probe, we have tagged the chromosome of *E. coli* strain MG1655, adjacent to AraC regulated genes, and determined the relative cellular locations by fluorescence microscopy. We show that AraC-regulated genes, within different macrodomains, do not co-localise in the cell. However, we show that the *araFGH* operon, which is near to the replication terminus, is spatially repositioned upon induction of transcription. This was particularly evident in dividing cells, where it was observed that induction of transcription facilitated separation of newly-replicated sister chromatids.

## Methods

### Bacterial strains, plasmids and growth conditions

All bacterial strains and plasmids used in this study are listed in Additional file 1. For microscopy experiments, strains were grown in M9 minimal media, supplemented with 0.3% fructose and 0.1% casamino acids, at 23 °C for 24 h. Cultures were diluted 1:50 into fresh media and grown for a further 5–6 h until OD_650_ reached approximately 0.1. For cultures supplemented with sugars, a final concentration of 0.3% of the required sugar was added to the culture for 1 min before slides were prepared [15]. For cultures supplemented with erythromycin (20 μg/ml) or rifampicin (50 μg/ml), the antibiotics were added for 15 min prior to the addition of arabinose.

### Construction of plasmids for MalI FROS

pLER108, carrying the *malI::mcherry* fusion, is a derivative of pACYC184 and carries resistance to chloramphenicol and contains the *p15A* origin of replication. The *malI* promoter and gene were amplified from the plasmid pACYCMalI using oligo’s D63433 and D71192 (Additional file 2) and digested with enzymes HindIII and KpnI and ligated into HindIII and KpnI digested pLER101, creating pLER104. Into this plasmid, the *mCherry* gene, which had been amplified from pmCherry-N1 using oligos D71000 and D71001, was ligated on a KpnI - MfeI digested fragment, resulting in a *malI:mCherry* gene fusion. This fusion was amplified using oligos D71850 and D72002 and the fragment cut with NsiI and HindIII was ligated into pJW15Δ100 to replace the *malI* promoter with the *melR* promoter, creating pLER105. Oligos D77566 and D77567 were used to amplify the promoter and fusion, the fragment was digested with HindIII and MfeI and ligated into pLER101, creating pLER108.

An array of MalI binding sites was created using the iterative PCR based method described by Lau et al.*,* 2003 [16]. Briefly, MalI binding sites were incorporated into pUC19 using oligos with a 5′ end consisting of a MalI binding site and a 3′ end consisting of pUC19 homology (D71689 and D71690). Thus, using pUC19 as a template for PCR, these oligos were used to create a product that could be ligated to form a plasmid containing 2 MalI binding sites, flanked on one side by an XbaI restriction site and on the other side by NheI and HindIII restriction sites. This plasmid was used to generate both vector, by digesting with NheI and HindIII, and insert, by digesting with XbaI and NheI: ligation of these two products generated a new plasmid that contained 4 MalI binding sites separated by a hybrid XbaI/NheI site This was repeated until there were 20 MalI binding sites (MalO), creating pUCMal20.

### Construction of gene doctoring donor plasmids

Gene doctoring donor plasmids were derived from pJB32 [17]. These carry the 22 *lac* operator sites (LacO array) or MalO array and a kanamycin cassette, flanked by 500 bp regions of homology from both sides of the insertion site, adjacent to either the *araBAD, araJ* or *araFGH* for MalO, or adjacent to either *araBAD, araJ* or *dps* for LacO. Oligonucleotides were designed to amplify 500 bp upstream of each insertion site, (Additional file 3) inserting a MfeI site upstream and a XmaI site downstream. This fragment was digested with MfeI and XmaI and ligated into MfeI and XmaI digested pJB32. Oligonucleotides were also designed to amplify 500 bp downstream of each insertion site, and insert a NheI site upstream and SacI site downstream. This product was digested with NheI and SacI and ligated with vector prepared from the previous ligation, digested with the same enzymes. Into the resulting plasmids, the LacO and MalO arrays were inserted: the LacO array was digested from pPM301 on a BglII/NheI fragment and the MalO array was digested from pUCMal20 on an XhoI/NheI. The plasmids that were generated are listed in Additional file 2.

### Chromosomal recombination

Gene doctoring was used to make chromosomal modifications using the donor plasmids constructed as described above [18]. MalO arrays were inserted into the chromosome of MG1655, LacO arrays were inserted into strain DL02. For two colour analysis, the MalO array was inserted into strains already harbouring a LacO array. Candidates were screened for the insert by colony PCR using oligonucleotides designed to bind to the chromosome outside the homology regions. The kanamycin resistance cassette was removed from the chromosome using flippase recombinase (FLP) expressed from plasmid pCP20 [19]. The resulting strains are listed in Additional file 1.

### Microscopy

Bacterial cultures were grown for 24 h at 23 °C [20] with aeration in M9 minimal salts media supplemented with 0.3% fructose, 2 mM MgSO_4_, 0.1 mM CaCl_2_, 0.1% casamino acids and, if necessary, 17.5 μg/ml chloramphenicol. Cultures were diluted 1:50 and grown under the same conditions until cultures reached OD_650_ 0.1. 1 ml of culture was removed and washed 3 times with PBS then resuspended in 20 μl Hoechst 33,258 solution containing 5 μg/ml Hoechst 33,258 in PBS containing 40% glycerol. 5 μl were loaded onto poly-L-lysine coated slides and a cover slip applied. Slides were imaged using a Nikon Eclipse 90i microscope, Nikon Intensilight C-HGFI lamp, Hamamatsu ORCA ER camera (1344 × 1024 pixels, pixel size 6.45 μm) and Nikon Plan Apo VC 100× Oil immersion lens (Numerical Aperture 1.4), with a final optical magnification of 100×. A DAPI filter set was used for visualising the Hoechst 33,258 stained nucleoid, FITC filter set for GFP and TxRed filter set for mCherry. Cells were also imaged using brightfield. Microscopy was carried out at room temperature, within 30 min of slides being prepared.

### Analysis of microscopy

Microscope images were analysed using Image J software. To determine the position of foci within cells, the measuring function was used to measure both the length of the cell and the distance from the focus to the nearest pole. The position of the focus within the cell was then calculated and is presented relative to the length of the cell, which was set at an arbitrary value of 1. For cells containing two foci, the focus nearest to a pole was designated as the ‘1st of 2 foci’, and the distance from the focus to the nearest pole was measured. The distance from the ‘2nd of 2 foci’ to the same pole was then measured. Measurements were taken from at least 300 cells. Where the data are presented on a scatter plot (Fig. 5), the relative position of the first focus is plotted on the x axis, and the relative position of the second focus is plotted on the y axis. To analyse co-localisation, the position of each of the two foci was measured using NIS elements software (Nikon), which provided a measurement in μm. To determine if the data, pre and post arabinose induction were significantly different, ANOVA or T-tests were done suing Excel software. Cells that had multiple foci of the same colour were not included in this analysis.

## Results

### MalI as a FROS reporter system

**Fig. 1:**
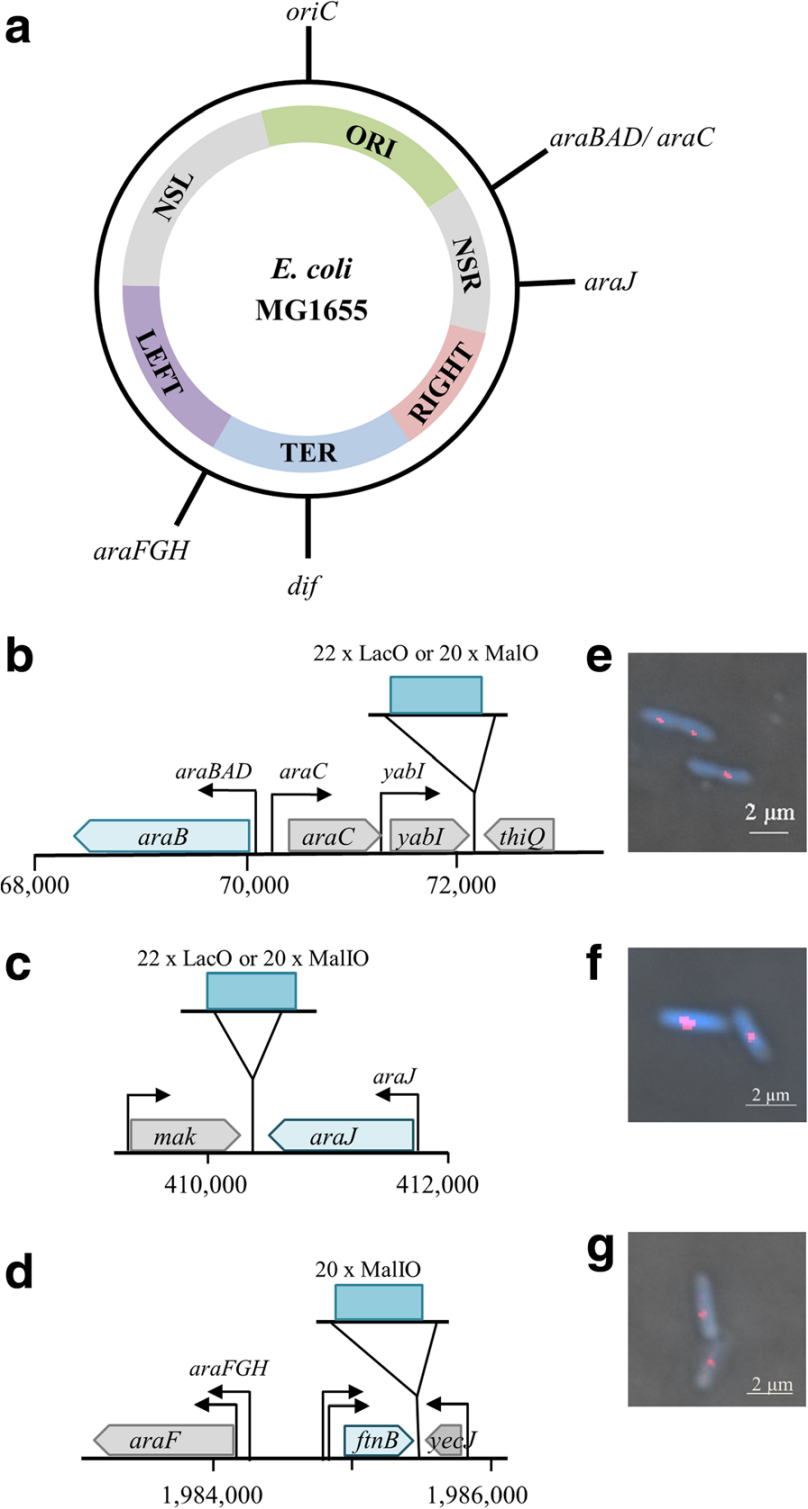
FROS Tagging AraC regulated promoters. **a** A schematic representation of the circular chromosome of *E. coli* strain MG1655. Macrodomains of chromosome organisation are displayed and the origin of replication (*oriC*) and the region of termination (*dif*) are highlighted [3, 24]. The positions of AraC-regulated promoters and the *dps* gene on the circular chromosome are shown. Multiple *lac* operators (LacO) or MalI DNA binding sites (MalO) were inserted adjacent to *araBAD* (**b**), adjacent to *araJ* (**c**) or adjacent to *araFGH* (**d**). Panels (**e**), (**f**) and (**g**) show examples of fluorescent foci derived from MalI:mCherry binding to a 20 MalO array inserted adjacent to *araBAD*, *araJ* and *araFGH* respectively. The images shown are merged images of MalI;mCherry foci and Hoechst 33,258 stained chromosomes

Several methodologies have been employed to examine nucleoid structure, one of which is the use of Fluorescent Reporter Operator Systems (FROS) [8–12]. Typically, a DNA binding protein (Reporter), fused to a fluorescent tag, is targeted to an array of DNA target sites (Operators), with resulting fluorescent foci being visualised by microscopy. The *E. coli* K-12 MalI protein is a transcription repressor associated with the Mal operon, and is a member of the GalR/LacI family of DNA binding proteins [13, 14]. The *malI* gene is located on its chromosome, convergent to the *malXY* operon. When expressed, MalI binds to a 16 bp target site at both the *malI* and *malXY* promoters to repress transcription. To generate an array of MalI binding sites, we used an iterative PCR procedure, followed by a cloning approach to build the required number of DNA binding sites in a plasmid. Hence, 20 DNA sites for MalI were incorporated into the MalI operator array (MalO), which was then targeted to specific positions in the chromosome of *E. coli* strain MG1655 using the gene doctoring recombineering method [18]. The array was inserted at three chromosomal targets: adjacent to the *araBAD, araJ* and *araFGH* promoter regions (Fig. 1a – d and Additional file 4). The *araJ* and *araBAD* loci are situated on the *E. coli* K-12 chromosome within the non-structured right domain, more than 1 Mbp away from the *araFGH* operon which is within the Ter macrodomain. Hence, the co-localisation and movement of commonly regulated genes within the same domain, and within different domains, could be examined.

To generate a MalI:mCherry fusion protein, the *malI:mCherry* gene fusion was cloned downstream of the *melR* promoter in plasmid pACYC184, creating plasmid pLER108. This resulted in constitutive, low level expression of MalI:mCherry. To examine the DNA binding efficiency and fluorescence derived from the fusion protein, wildtype MG1655 cells, and cells carrying the MalO array situated at the *araBAD, araJ* and *araFGH* loci were transformed with pLER108. Fluorescence derived from cells in the mid-logarithmic phase of growth was examined using epifluorescence microscopy. In the absence of a MalO array, there are no visible foci and the background fluorescence in the cell was negligible (Additional File 5). The images in Fig. 1e – g show MG1665 cells that contain the MalO array at the *araBAD, araJ* and *araFGH* loci respectively. Foci derived from MalI:mCherry bound at the MalO array are clearly observed in each case. Thus MalI:mCherry bound to the MalO array is functional as a reporter:operator system for FROS.

### Modification of the LacI:GFP FROS reporter system and comparison with MalI:mCherry

**Fig. 2:**
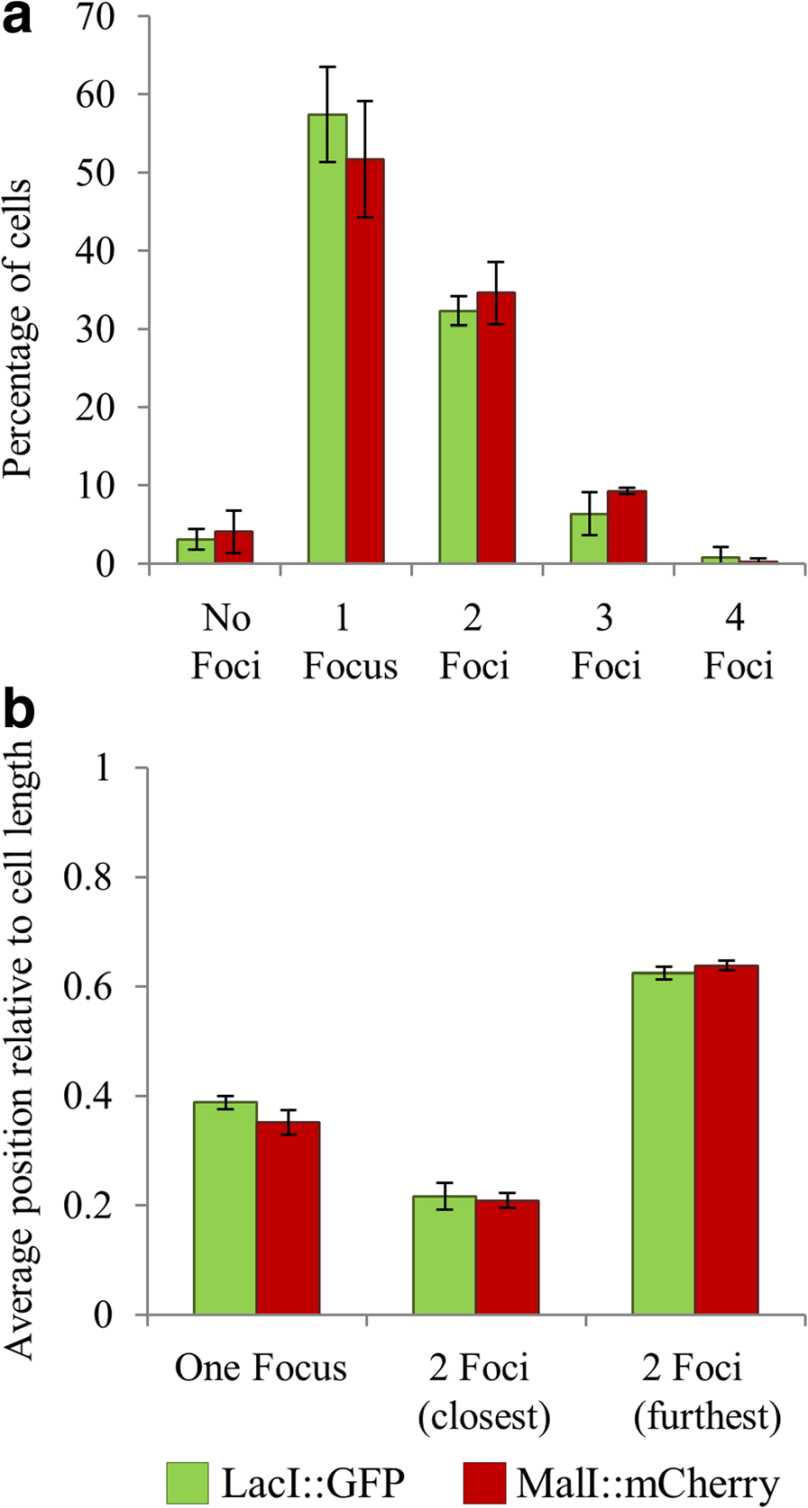
Comparison of MalI and LacI FROS probes. **a** The number of fluorescent foci were counted in 300 individual cells, grown in M9 minimal medium without arabinose, containing either the MalO or LacO arrays inserted adjacent to *araBAD*. **b** The distance to the nearest cell pole for foci in cells that contained 1 or 2 foci. For 2 foci analysis, the distance of the focus nearest to a pole was measured (closest) and the distance of the second focus to the same pole was then measured (furthest). Error bars represent the standard deviation

Previous studies have visualised the LacI:GFP fusion protein bound to a large chromosomal target array containing 256 copies of the LacI DNA binding site [11]. Since we demonstrated that MalI foci could be readily visualised bound to an array of 20 MalI DNA binding sites, we sought to reduce the number of LacI binding sites in an array. In our previous work, we observed LacI binding to co-localised plasmids, corresponding to approximately 25 *lacI* target sites (plasmid copy number of 5: each plasmid containing 5 *lacI* DNA binding sites) [6]. Thus, we generated a LacO array, consisting of 22 *lacI* binding sites, which we introduced at the *araBAD* and *araJ* loci in MG1655 cells harbouring a *lacI:gfp* chromosome fusion at the natural *lacI* loci (Fig. 1b and c). Cells harbouring the LacIO or MalO arrays were then examined using epifluorescence microscopy and the number of foci and the position of the foci relative to the length of the cell was determined. The results in Fig. 2a, for the *araBAD* locus, show that the distribution of cells containing foci was comparable when the number of foci derived from the MalI and LacI FROS probes was counted. One focus was observed in the majority of cells, with 2 foci observed in a large proportion of cells which were actively undergoing chromosome segregation. Based on these data, the average number of foci per cell was calculated to be approximately 1.4, which is consistent with our previous measurements of the the average numbers of chromosomes per cell in these growth conditions. When the average position of the foci from cells containing either 1 or 2 foci was then measured, with respect to total cell length, the data derived from the two FROS probes was comparable (Fig. 2b). This indicates that the *araBAD* locus is similarly positioned within the cell when tagged with either the MalI or LacI FROS reporter systems.

### Co-localisation of AraC regulated promoters

**Fig. 3:**
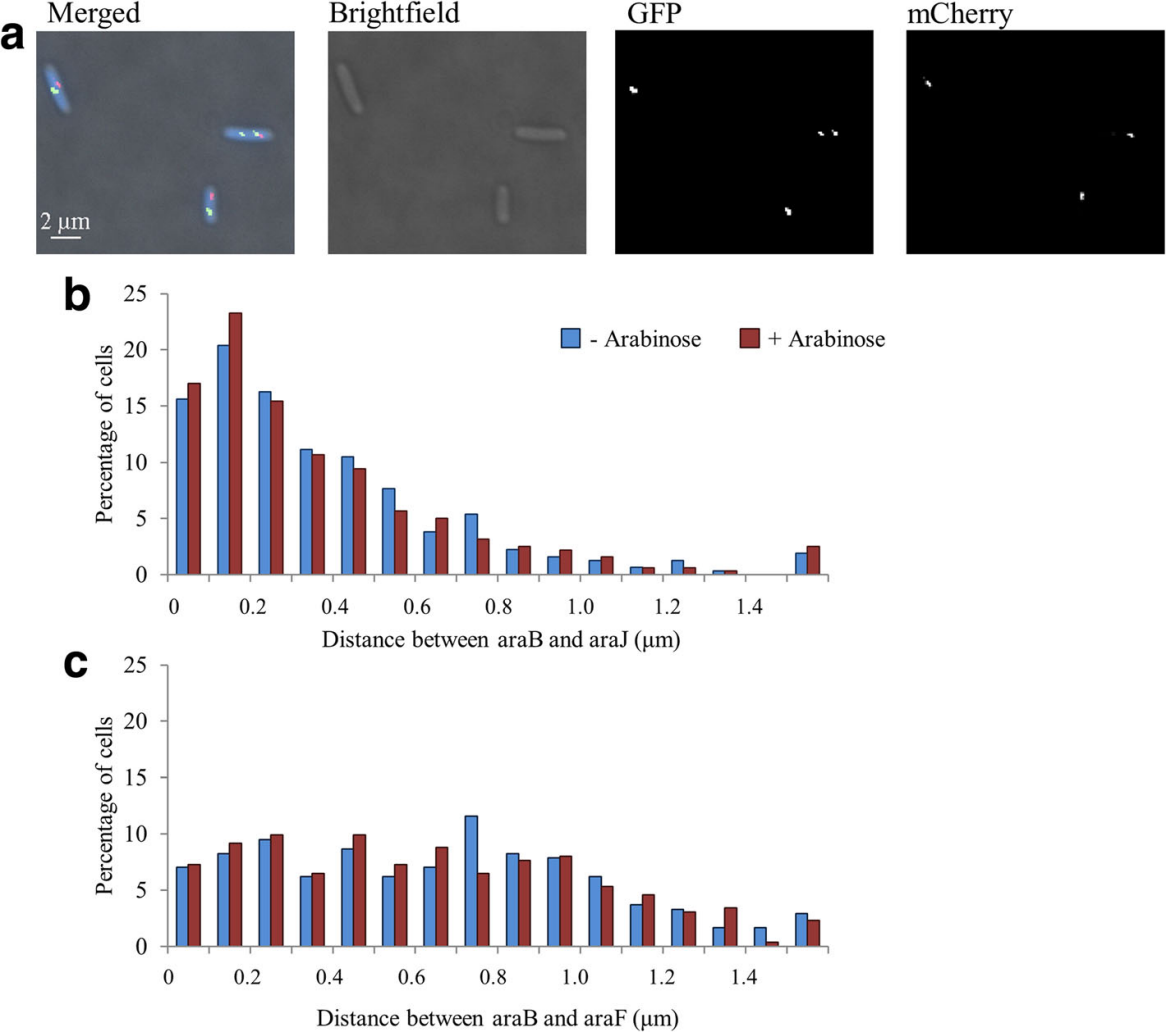
Colocalisation of genes regulated by AraC. **a** The figure shows a dual fluorescence image of strain LR31, carrying a LacO array at *araBAD* and a MalO array at *araJ* stained with Hoechst 33,258. **b** and **c** The bar charts show the distance between two distal chromosomal locations, each independently tagged with different FROS reporters. **b** Distance measurements between the *araBAD* locus, tagged with a LacO array, and the *araJ* locus, tagged with a MalO array, were calculated in 300 individual cells. Absolute distances between the two chromosomal locations in the presence and absence of the inducer, arabinose, are plotted. **c** Distance measurements between the *araBAD* locus, tagged with a LacO array, and the *araFGH* locus, tagged with a MalO array, were calculated in 300 individual cells. Absolute distances between the two chromosomal locations in the presence and absence of the inducer, arabinose, are plotted

To assess whether AraC regulated promoters co-localised, strains of *E. coli* were generated that contained a LacI:GFP FROS probe adjacent to the *araBAD* promoter and a MalI:mCherry FROS probe at either the *araJ* or *araFGH* promoters. Individual cells of these strains, grown either in the presence or absence of arabinose, were visualised using fluorescence microscopy (Fig. 3a). Cells containing different numbers of each fluorescent cluster were observed, containing clear and distinct foci derived from GFP and mCherry. To calculate the distance between the MalI:mCherry foci and the LacI:GFP foci, the distance from the GFP focus to the closest pole was measured, and subtracted from the distance of the mCherry focus to the same pole. Hence, the distances between the *araBAD* and *araJ* promoters, and the *araBAD* and *araFGH* promoters were calculated in >500 individual cells, grown in the presence or absence of arabinose (Fig. 3b and c). The principal observation was that the distance between the foci varied substantially throughout the population, but this did not significantly alter upon addition of arabinose. The range of distances between *araBAD* and *araJ* probes (average 0.37 μm) was less than between the *araBAD* and *araFGH* probes (average 0.64 μm). This was expected since *araBAD* and *araJ* are located within the same macrodomain, whereas the *araBAD* and *araFGH* are in different domains. Thus, unlike previously reported with GalR regulated promoters [5], the AraC regulated promoters do not appear to co-localise in the bacterial nucleoid.

### Location and dynamics of AraC regulated promoters

**Fig. 4:**
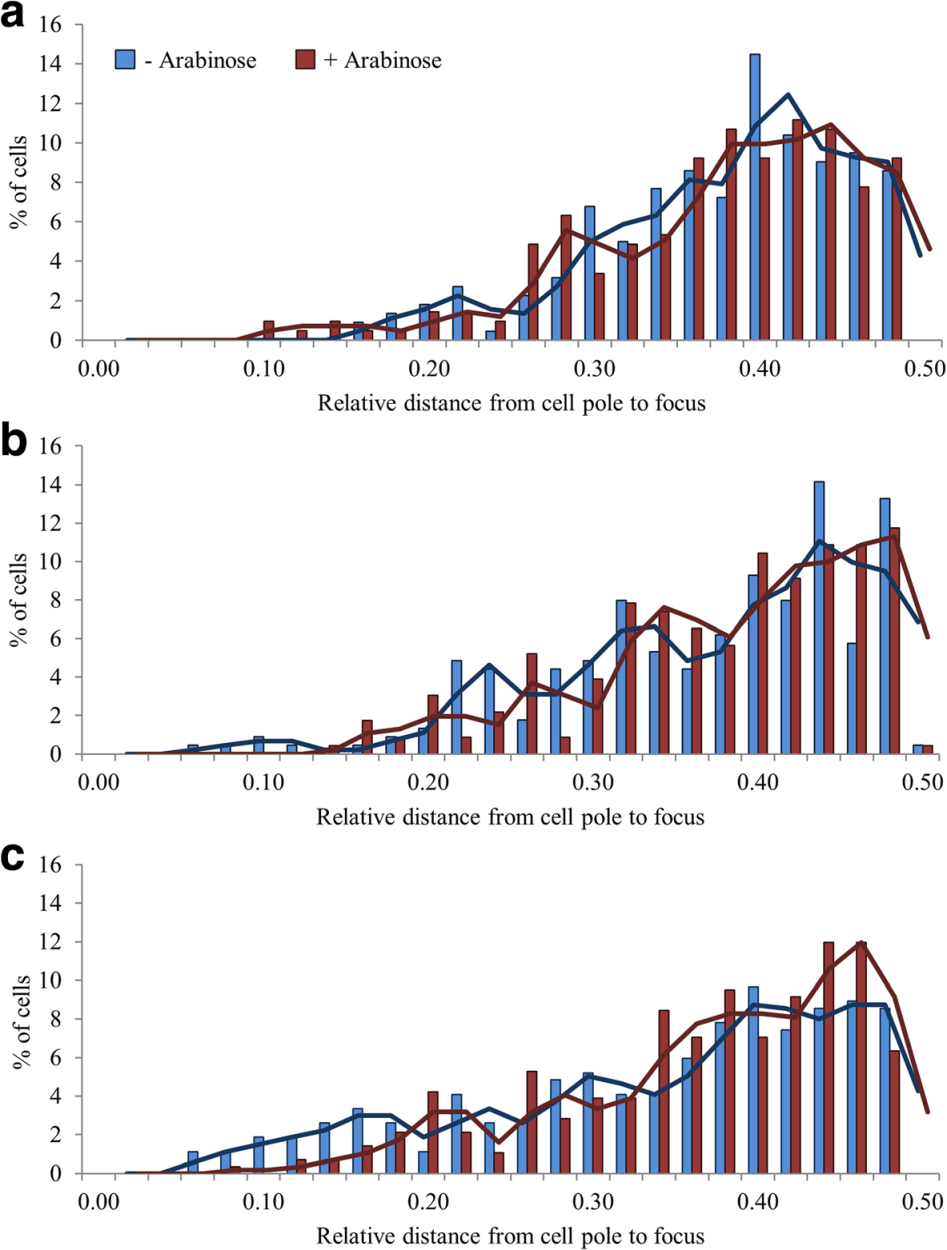
Relative cellular location of AraC regulated promoters in the presence and absence of inducer. The distances between fluorescent foci and the nearest cell pole was measured in 300 individual cells containing a single fluorescent foci derived from FROS probes adjacent to (**a**) *araBAD*, strain LR06, (**b**) *araJ*, strain LR39 and (**c**) *araFGH*, strain LR38. Distances are plotted, relative to cell length, in the presence and absence of the inducer, arabinose. For these experiments, *araBAD* and *araJ* were tagged with a LacO array and *araFGH* was tagged with a MalO array. The experiment was repeated on 3 separate occasions, with the same outcome observed. Associated *P*-values for uninduced compared to induced cells are: for *araBAD,* 0.556; for *araJ,* 0.252; and for *araF*, 0.005

Since AraC-regulated promoters did not appear to co-localise, next we examined whether individual promoter regions were repositioned upon induction. To do this, *E. coli* strains containing LacI:GFP FROS probes at the *araBAD* and *araJ* loci, and the MalI:mCherry FROS probe at the *araFGH* locus, were grown in the presence or absence of arabinose. Cells were analysed by fluorescence microscopy, and individual, non-dividing cells containing a single fluorescent focus were analysed. The distance from each focus to the nearest cell pole was measured, and this value was divided by the total cell length, thereby providing a position relative to total cell length (Fig. 4). Foci derived from the FROS probes positioned near to the *araBAD* and *araJ* regions did not reposition when the promoters were induced by arabinose (Fig. 4a and b). However, in a small proportion of the cells, the FROS probe adjacent to the *araFGH* operon relocated away from the cell pole towards the centre of the cell upon induction (Fig. 4c: redistribution of cells with a focus between 0.05 and 0.18 upon induction with arabinose).

**Fig. 5:**
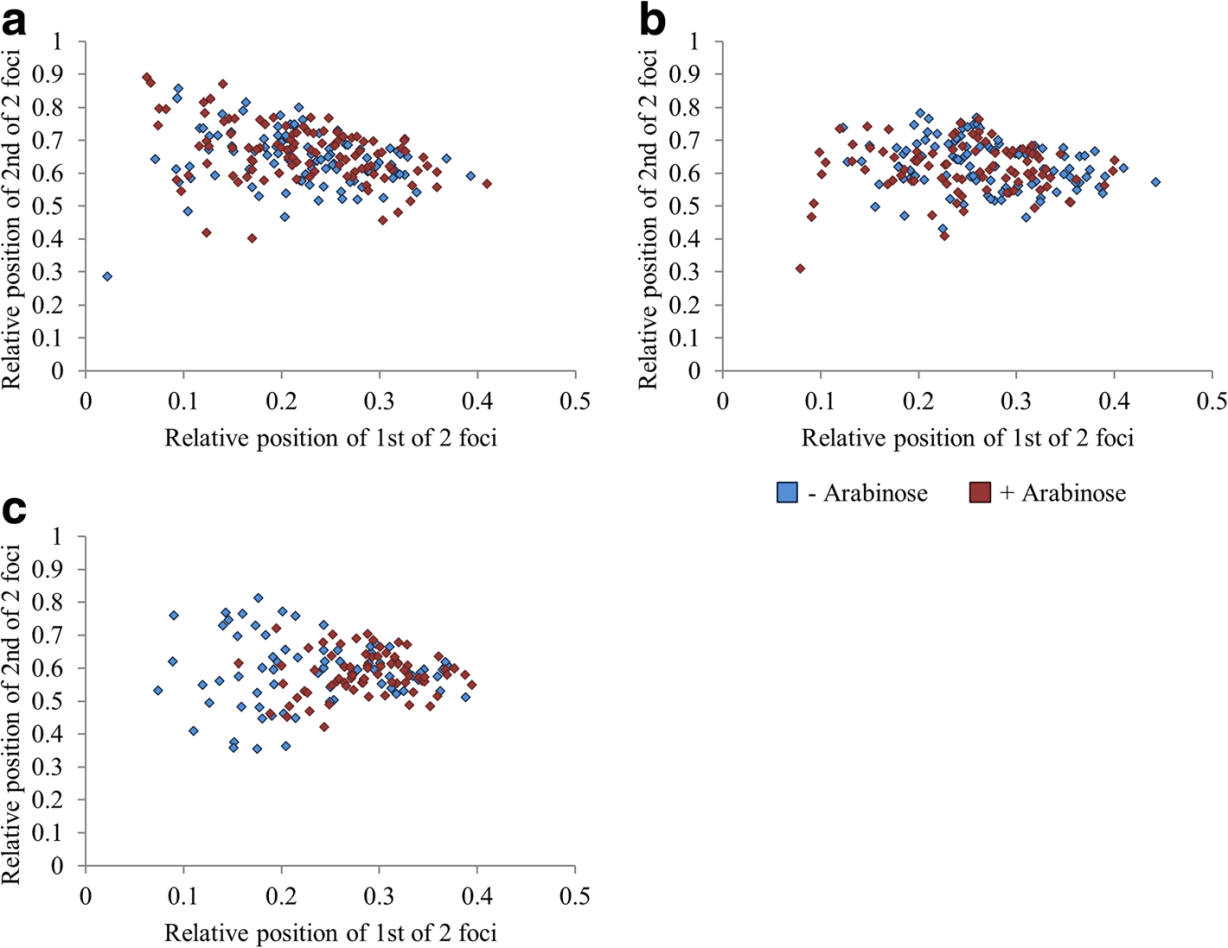
Relative positions of two fluorescent foci in cells containing FROS probes adjacent to different AraC-regulated promoters in the presence and absence of inducer. The distances between foci were measured in 300 individual cells containing two fluorescent foci derived from FROS probes adjacent to (**a**) *araBAD*, strain LR06, (**b**) *araJ*, strain LR39 and (**c**) *araFGH*, strain LR38. The distance between the focus closest to a cell pole and that cell pole was first measured and calibrated to the relative cell length (1st of 2 foci). The distance from the cell pole to the second focus was then measured relative to cell length. The cellular position of the second focus was then plotted against the position of the first focus from cells grown in the presence and absence of the inducer, arabinose. For these experiments, *araBAD* and *araJ* were tagged with a LacO array and *araFGH* was tagged with a MalO array

A similar relocation of the *araFGH* locus was observed in cells containing two foci. Fig. 5 shows the relative position of each of the two foci associated with an AraC-regulated promoter. When grown in the presence or absence of arabinose, no discernible repositioning was observed with the probe at the *araBAD* or *araJ* loci. However, for the *araFGH* locus, the focus closest to the cell pole repositioned, with an overall movement away from the cell pole. Thus, the two foci were repositioned relative to each other upon arabinose induction.

**Fig. 6:**
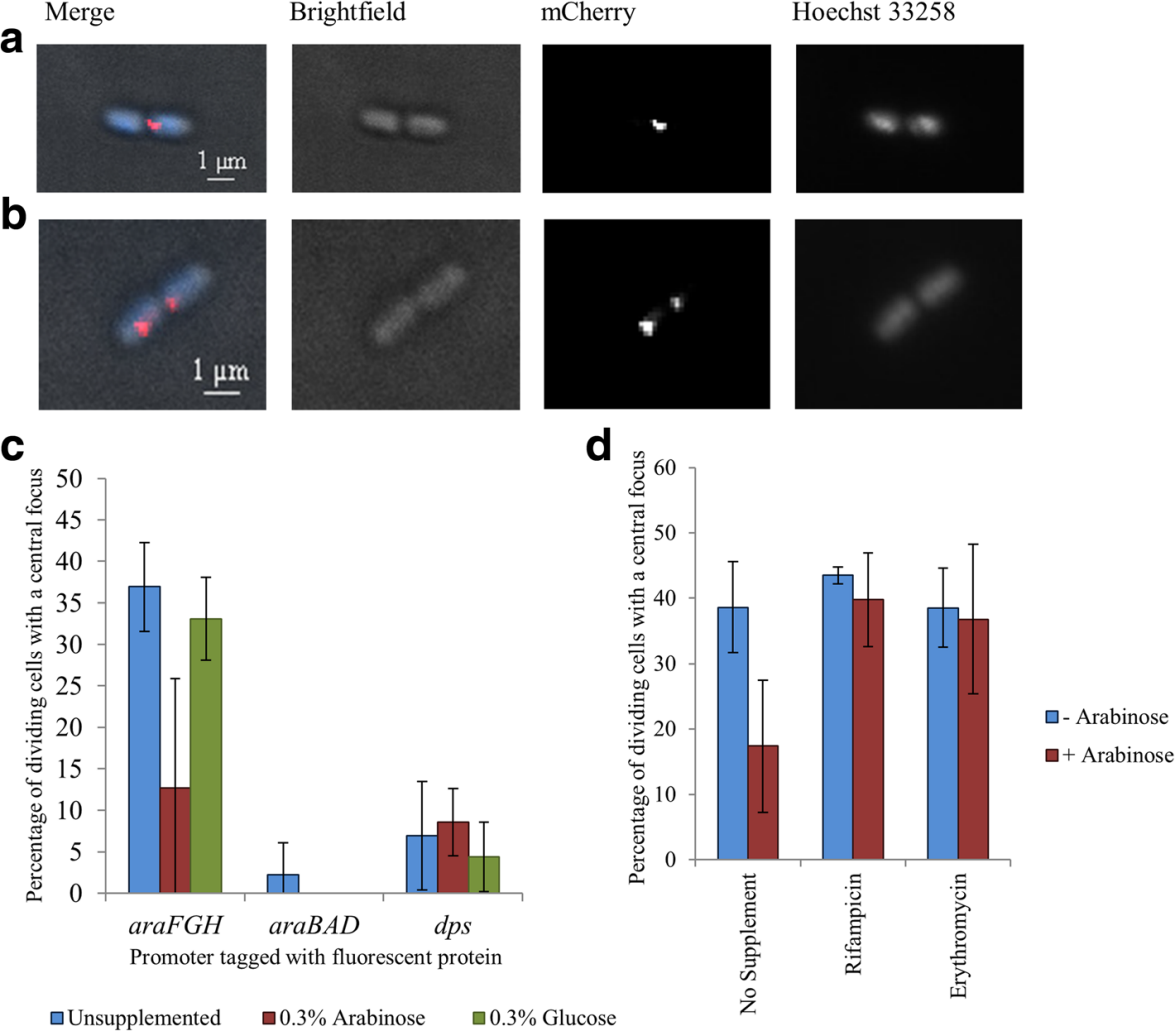
Gene expression drives chromosome separation. The number of fluorescent foci in cells at the point of division were counted in the presence and absence of the inducer, arabinose. Dividing cells were defined as having two separate nucleoids when stained with Hoechst 33,258 but which were not separate cells when viewed under brightfield microscopy. Cells containing a 20 MalO array adjacent to the *araFGH* locus, predominantly contained either a single centrally located focus (**a**) or two distinct foci (**b**). **c** The number of foci in 300 individual cells were counted, from bacterial cultures grown in minimal medium, supplemented with 0.3% arabinose, or supplemented with 0.3% glucose. For this experiment, *araBAD* and *dps* were tagged with a LacO array and *araFGH* was tagged with a MalO array. For each position, the percentage of dividing cells containing a single central focus is plotted. The values for the *araBAD* tagged strain, induced with arabinose and glucose, were 0%. **d** The impact of inhibiting the processes of transcription of translation on the number of cells containing a single focus derived from a MalO array adjacent to *araFGH*. Growing cultures were supplemented with rifampicin or erythromycin prior to induction with arabinose. The number of cells containing a single focus, from 300 individual cells, is plotted and the error bars represent the standard deviation

To examine further the movement of the *araFGH* locus upon induction, we studied the position of foci in cells at the point of division (Fig. 6a and b). These dividing cells were defined as cells that had two separate nucleoids when stained with Hoechst 33,258 but which did not appear to be two distinct, separate cells when viewed by brightfield microscopy. Such cells accounted for 5–15% of all cells, and in uninduced conditions, approximately 35% of these contained a single *araFGH* focus (Fig. 6a & c). In contrast, when the FROS probe was positioned at the *araBAD* locus, which is more proximal to the origin of replication, very few cells had a single focus (2%), with 98% of cells containing at least 2 foci. In conditions of growth supplemented with glucose or arabinose, no change in the number of *araBAD* foci in each individual cell was observed. Similarly, no change in the number of fluorescent foci in each individual cell was observed when the FROS probe was located adjacent to the *dps* promoter that was used as a control region of the chromosome, unaffected by arabinose. However, at the *araFGH,* there was a clear reduction in the number of cells containing only one foci locus in the presence of arabinose, but not glucose. The observed shift from 37% of the population containing a single focus to 13% upon induction suggests that expression of the *araFGH* operon assists separation of newly replicated sister chromatids. To test this, cultures were supplemented with arabinose, to induce expression of the *araFGH* operon, and either rifampicin: to inhibit transcription, or erythromycin: to inhibit translation. In both cases, the addition of the inhibitors prevented the separation of foci (Fig. 6d). Treatment of cells with these antibiotics is likely to impact upon the transcription and translation of every gene within the cell. Thus to confirm that the processes of gene expression at the *araFGH* operon are directly responsible for our observations, direct targeting of the individual DNA promoter elements and ribosome binding sites of the promoters driving expression of fluorescent protein fusions would be necessary. Nevertheless, our data provide compelling evidence that chromosome separation at the *araFGH* locus is enhanced by the processes of gene expression.

## Discussion

The aim of this study was to investigate possible transcription factor clustering in a bacterial nucleoid and to investigate changes in response to transcription. Hence, we sought to visualise nucleoid re-organisation and identify co-localisation of distant loci upon expression of commonly regulated genes. To facilitate this, we developed and validated a new fluorescent reporter-operator system, based on the *E. coli* transcription repressor protein, MalI, which was fused to the mCherry fluorescent protein. In combination with a LacI:GFP reporter, we tagged the chromosome of *E. coli* strain MG1655, with MalI or LacI DNA operator binding site arrays, adjacent to genes that are regulated by the AraC protein, so that the cellular location and transcription induced spatial repositioning of these commonly regulated genes could be monitored. We observed that AraC-regulated genes, located within the same nucleoid domain, or within different domains, do not co-localise in the cell. This is in contrast to what was found with GalR-regulated promoters, which are located within different domains, yet co-localise [5]. We speculate that the ability of GalR to tetramerise may be a driving force in enabling the GalR co-regulated regions to co-localise.

A second finding of this study was that induction of expression of a transcription unit near to the terminus of DNA replication resulted in enhanced separation of newly replicated chromosomes at that locus. We found that this was dependent on both transcription and translation, as inhibition of either prevented separation. We assume that the act of transcription is the driving force behind this observation, since transcription and translation are often coupled in bacteria [21], We suppose that transcription induced supercoiling may drive chromosome separation by enhancing the process of decatenation [22, 23], which is feasible since decatenation is facilitated by topoisomerase enzymes, and is thus impacted by DNA supercoiling.

## Conclusion

We have developed resources that facilitate two colour FROS analysis of regions of the chromosome within *Escherichia coli* cells. Our investigations indicate that distal regions on the linear chromosome that are regulated by transcription regulator AraC do not colocalise in the folded nucleoid. Our explorations do however, suggest a role for transcription in facilitating chromosome separation post replication.

## Additional files

Additional file 1: Strains used in this study. (DOCX 21 kb)

Additional file 2: Plasmids used in this study. (DOCX 23 kb)

Additional file 3: DNA oligonucleotides used in this study. (DOCX 20 kb)

Additional file 4: Schematic diagram to show the insertion sites of FROS operators adjacent to (a) *araBAD*, (b) *araFGH* and (c) *mntH*. (DOCX 74 kb)

Additional file 5: MalI:mCherry expressed from plasmid pLER108 in MG1655. (DOCX 325 kb)

## Abbreviations

FLP: Flippase recombinase
FROS: Fluorescent Reporter-Operator System
