# Supplementary material for: Development of a new fluorescent reporter:operator system: location of AraC regulated genes in Escherichia coli K-12
Source: BMC Microbiol. 2017 Aug 3;17:170. doi: 10.1186/s12866-017-1079-2 (PMC5543585; doi:10.1186/s12866-017-1079-2)
Supplement: Supplementary file 1 — Strains used in this study. (DOCX 21 kb) [file 12866_2017_1079_MOESM1_ESM.docx]

**Strains used in this study**

| Strain | Description | Origin |
| --- | --- | --- |
| MG1655 | *F- λ- ilvG rfb-50 rph-1* | [[1](#_ENREF_1)] |
| DL02 | MG1655 derivative with *lacI::gfp* inserted onto the chromosome at the natural *lacI* locus | [[2](#_ENREF_2)] |
| LR06 | Derivative of DL02 in which 22 *lac* operators have been inserted adjacent to *araBAD* and the *kan* cassette has been removed | This study |
| LR17 | Derivative of MG1655 in which 20 MalI binding sites have been inserted adjacent to *araBAD* and the *kan* cassette has been removed | This study |
| SXB4 | Derivative of DL02 in which 22 *lac* operators have been inserted adjacent to *dps* and the *kan* cassette has been removed | This study |
| LR31 | Derivative of LR06 in which 20 MalI binding sites have been inserted adjacent to *araJ* and the *kan* cassette has been removed | This study |
| LR38 | Derivative of MG1655 in which 20 MalI binding sites have been inserted adjacent to *araFGH* and the *kan* cassette has been removed | This study |
| LR39 | Derivative of DL02 in which 22 *lac* operators have been inserted adjacent to *araJ* and the *kan* cassette has been removed | This study |
| LR48 | Derivative of LR06 in which 20 MalI binding sites have been inserted adjacent to *araFGH* and the *kan* cassette has been removed | This study |

References

[1] Blattner FR, Plunkett G, 3rd, Bloch CA, Perna NT, Burland V, Riley M, et al. The complete genome sequence of Escherichia coli K-12. Science. 1997;277:1453-62.

[2] Lee DJ, Bingle LE, Heurlier K, Pallen MJ, Penn CW, Busby SJ, et al. Gene doctoring: a method for recombineering in laboratory and pathogenic Escherichia coli strains. BMC microbiology. 2009;9:252.
