# Supplementary material for: Development of a new fluorescent reporter:operator system: location of AraC regulated genes in Escherichia coli K-12
Source: BMC Microbiol. 2017 Aug 3;17:170. doi: 10.1186/s12866-017-1079-2 (PMC5543585; doi:10.1186/s12866-017-1079-2)
Supplement: Supplementary file 2 — Plasmids used in this study. (DOCX 23 kb) [file 12866_2017_1079_MOESM2_ESM.docx]

**Plasmids used in this study**

| Plasmid | Description | Origin |
| --- | --- | --- |
| pPM301 | pUC19 derivative containing 22 *lac operators* (Amp^R^) | P.McGlynn (unpublished) |
| pACYC184 | Cloning vector (Tet^R^ Cm^R^) | [[1](#_ENREF_3)] |
| pJB32 | Donor plasmid for gene doctoring used for inserting *plac GFP* onto the chromosome adjacent to *araBAD* (Kan^R^ Amp^R^) | [[2](#_ENREF_4)] |
| pCP20 | Temperature sensitive plasmid encoding FLP recombinase (Amp^R^ Cm^R^) | [[3](#_ENREF_5)] |
| pUC19 | Coloning vector (Amp^R^) | [[4](#_ENREF_6)] |
| pUCMal20 | pUC19 containing 20 MalI DNA sites (Amp^R^) | This study |
| pmCherry-N1 | Plasmid carrying mCherry | ClonTec |
| pJW15Δ100 | Plasmid for fusing genes to the *melR* promoter (Amp^R^) | [[5](#_ENREF_7)] |
| pACYCMalI | Derivative of pACYC184 containing *malI* | [[6](#_ENREF_8)] |
| pLER101 | Derivative of pACYC184 containing LacI fused to GFP under the control of the *lacI* promoter (Cm^R^) | [[7](#_ENREF_9)] |
| pLER104 | Derivative of pLER101 containing MalI fused to mCherry under the control of the *malI* promoter | This study |
| pLER105 | Derivative of pJW15 containing MalI::mCherry fused to *melR* promoter | This study |
| pLER108 | Derivative of pLER105 containing MalI fused to mCherry under the control of the *melR* promoter | This study |
| pLR8 | Derivative of pJB32 containing 22 *lac* operators and homology regions for insertion adjacent to *araBAD* | This study |
| pLR19 | Derivative of pJB32 containing 20 MalI binding sites and homology regions for insertion adjacent to *araBAD* | This study |
| pLR25 | Derivative of pJB32 containing 22 *lac* operators and homology regions for insertion adjacent to *araJ* | This study |
| pLR24 | Derivative of pJB32 containing 22 MalI binding sites and homology regions for insertion adjacent to *araJ* | This study |
| pLR17 | Derivative of pJB32 containing 20 MalI binding sites and homology regions for insertion adjacent to *araFGH* | This study |
| pSB6 | Derivative of pJB32 containing 22 *lac* operators and homology regions for insertion adjacent to *dps* | This study |
|  |  |  |

**References**

[1] Chang AC, Cohen SN. Construction and characterization of amplifiable multicopy DNA cloning vehicles derived from the P15A cryptic miniplasmid. Journal of bacteriology. 1978;134:1141-56.

[2] Bryant JA, Sellars LE, Busby SJ, Lee DJ. Chromosome position effects on gene expression in Escherichia coli K-12. Nucleic Acids Res. 2014.

[3] Cherepanov PP, Wackernagel W. Gene disruption in Escherichia coli: TcR and KmR cassettes with the option of Flp-catalyzed excision of the antibiotic-resistance determinant. Gene. 1995;158:9-14.

[4] Yanisch-Perron C, Vieira J, Messing J. Improved M13 phage cloning vectors and host strains: nucleotide sequences of the M13mp18 and pUC19 vectors. Gene. 1985;33:103-19.

[5] Kahramanoglou C, Webster CL, El-Robh MS, Belyaeva TA, Busby SJW. Mutational analysis of the Escherichia coli melR gene suggests a two-state concerted model to explain transcriptional activation and repression in the melibiose operon. Journal of bacteriology. 2006;188:3199-207.

[6] Lloyd GS, Godfrey RE, Busby SJ. Targets for the MalI repressor at the divergent Escherichia coli K-12 malX-malI promoters. FEMS microbiology letters. 2010;305:28-34.

[7] Sellars LE. Bacterial chromosome organisation and transcription. etheses:4994. 2014;School of Biosciences, University of Birmingham.
