# Supplementary material for: Development of a new fluorescent reporter:operator system: location of AraC regulated genes in Escherichia coli K-12
Source: BMC Microbiol. 2017 Aug 3;17:170. doi: 10.1186/s12866-017-1079-2 (PMC5543585; doi:10.1186/s12866-017-1079-2)
Supplement: Supplementary file 3 — DNA oligonucleotides used in this study. (DOCX 20 kb) [file 12866_2017_1079_MOESM3_ESM.docx]

DNA oligonucleotides used in this study (continued on next page)

| Name | Sequence (5’→3’) | Use |
| --- | --- | --- |
| *Oligonucleotides used for gene doctoring donor plasmids – Insert adjacent to araBAD* | | |
| D69231 | GCCGCAATTGCCGGGATTGAAACTGAACG | Upstream primer for amplification of the *thiQ* homology region. Carries MfeI site. |
| D69232 | GCCGCCCGGGCGACGCTTGCCGCGTCTTATC | Downstream primer for amplification of the *thiQ* homology region. Carries XmaI site. |
| D69234 | GCCGGCTAGCCATCAGGCAACCCCGCAC | Upstream primer for amplification of the *yabI* homology region. Carries NheI site. |
| D69233 | GCCGGAGCTCCTGAACATGCGTTGCATCAAC | Upstream primer for amplification of the *yabI* homology region. Carries SacI site. |
| D69747 | GTCGCACAGAACATCGG | Upstream primer for checking inserts adjacent to *araBAD* |
| D69748 | TCGCTGGTCATTTCTGAAG | Downstream primer for checking inserts adjacent to *araBAD* |
| *Oligonucleotides used for gene doctoring donor plasmids – Insert adjacent to araFGH* | | |
| D74949 | CGGCAATTGCTCTCAAATGAACCGCGA | Upstream primer for amplification of the *ftnB* homology region. Carries MfeI site. |
| D74950 | TAACCCGGGCAGATGAGGCAGCGG | Downstream primer for amplification of the *ftnB* homology region. Carries XmaI site. |
| D74951 | CAAGCTAGCTGTTTGAAGCAGCGG | Upstream primer for amplification of the *ypeC* homology region. Carries NheI site. |
| D74952 | GTCGAGCTCTGTCATATTATAAGCGC | Upstream primer for amplification of the *ypeC* homology region. Carries SacI site. |
| D75296 | AGGTATGGCAACCGCTGG | Upstream primer for checking inserts adjacent to *araFGH* |
| D75297 | TGCTGCGACAATGGCCG | Downstream primer for checking inserts adjacent to *araFGH* |
| *Oligonucleotides used for gene doctoring donor plasmids – Insert adjacent to araJ* | | |
| D75738 | GCTCAATTGTGCGCGGGATTATTTGCC | Upstream primer for amplification of the *araJ* homology region. Carries MfeI site. |
| D75739 | GCTCCCGGGATCATGCCTGATGCGACG | Downstream primer for amplification of the *araJ* homology region. Carries XmaI site. |
| D75740 | GCTGCTAGCGCGCCAATTGCCTACGTT | Upstream primer for amplification of the *mak* homology region. Carries NheI site. |
| D75741 | GCTGAGCTCATCGGCACGGGATGCG | Downstream primer for amplification of the *mak* homology region. Carries SacI site. |
| D76827 | TTCACCACTGCGCATTGCAGC | Upstream primer for checking inserts adjacent to *araJ* |
| D76828 | TTCAGAAGCAGTAGATGGCGCG | Downstream primer for checking inserts adjacent to *araJ* |
| *Oligonucleotides used for gene doctoring donor plasmids – Insert adjacent to* *dps* | | |
| D75742 | GCTCAATTGTGTGGTTCCTGCTACCG | Upstream primer for amplification of the *rhtA* homology region. Carries MfeI site. |
| D75743 | GCTCCCGGGGAGAAATTCTGCATGGTTATGC | Downstream primer for amplification of the *rhtA* homology region. Carries XmaI site. |
|  |  |  |
| D75744 | GCTGCTAGCGCTACTTTTCCTCTACACCG | Upstream primer for amplification of the *dps* homology region. Carries NheI site. |
| D75745 | GCTGAGCTCCCCCAGAGCTACACCG | Downstream primer for amplification of the *dps* homology region. Carries SacI site. |
| D76491 | TTTCGTCTGGGTTGTGCTGGC | Upstream primer for checking inserts adjacent to *dps* |
| D76492 | CGTTGTGGATGTCCAGCG | Downstream primer for checking inserts adjacent to *dps* |
| *Oligonucleotides used for cloning repressor protein::fluorescent protein tags into plasmids* | | |
| D71000 | CTGGGTACCATGGTGAGCAAGGG | Upstream primer for amplifying *mCherry* from plasmid pmCherry-N1. Carries a KpnI site |
| D71001 | CTGCAATTGCTAGAGTCGCGGCC | Downstream primer for amplifying *mCherry* from plasmid pmCherry-N1. Carries a MfeI site |
| D63433 | CGATAAGCTTCAAAACGTTTTATCAAATTTTAGTG | Upstream primer for amplifying *malI* from pACYCMalI. Carries a HindIII site |
| D71192 | TTAGGTACCTTTCGCTGCAATGAGCC | Downstream primer for amplifying *malI* from pACYCMalI. Carries a KpnI site |
| D72022 | CATGATGCATGCTACCGCCAAAAAAATAACC | Upstream primer for amplifying *malI::mCherry* from pLER104 without the *malI* promoter. Carries a NsiI site |
| D71850 | CATAAAGCTTCAATTGCTAGAGTCGCGG | Downstream primer for amplifying *malI::mCherry* from pLER104 without the *malI* promoter. Carries a NsiI site |
| D77566 | GTGAAGCTTCCGGGGATCCCGGGAAGA | Upstream primer for amplifying *malI::mCherry* under the control of the *melR* promoter from pLER105. Carries a HindIII site |
| D77567 | GTGCAATTGCTAGAGTCGCGGCCGCTA | Downstream primer for amplifying *malI::mCherry* under the control of the *melR* promoter from pLER105. Carries a MfeI site |
| *Oligonucleotides used for generating a multiple MalI DNA site array* | | |
| D71689 | CGAGTCGACACGT**GATAAAACGTTTTATC**AGGACTCTAGAGGATCCCCGGG | Primer for amplifying pUC19 introducing a MalI DNA site (bold) and SalI site (boxed). Carries a XbaI site (underlined) |
| D71690 | CGACTCGAGCATG**GATAAAACGTTTTATC***GCTAGC*TGCAAGCTTGGCGTAATCATGGTC | Primer for amplifying pUC19 introducing a MalI DNA site (bold), XhoI site (boxed) and NheI site (italics). Carries a HindIII site (underlined) |
