# Supplementary material for: Development of a new fluorescent reporter:operator system: location of AraC regulated genes in Escherichia coli K-12
Source: BMC Microbiol. 2017 Aug 3;17:170. doi: 10.1186/s12866-017-1079-2 (PMC5543585; doi:10.1186/s12866-017-1079-2)
Supplement: Supplementary file 4 — Schematic diagram to show the insertion sites of FROS operators adjacent to (a) araBAD, (b) araFGH and (c) mntH. (DOCX 74 kb) [file 12866_2017_1079_MOESM4_ESM.docx]

D69747

*ara araA araB araC yabI thiQ thiP tbpA*

(a)

(b)

(c)

D69748

HR1 HR2

*araH araG araH ftnB yecJ*

D75296

D75297

HR1 HR2

D76494

*glk yfeO ypeC mntH nupC*

D76493

HR2 HR1

**Schematic diagram to show the insertion sites of FROS operators adjacent to (a) *araBAD*, (b) *araFGH* and (c) *mntH***. For each site of insertion, a homology region of around 500 bp (HR1) was amplified from the chromosome on a MfeI-XmaI fragment and a second homology region of around 500 bp (HR2) was amplified from the chromosome on a NheI-SacI fragment. These fragments were cloned sequentially into plasmid pJB32. For insertion adjacent to *araBAD* primers D69231 and D69232 were used for HR1 and D69233 and D69234 were used for HR2. For insertion adjacent to *araFGH* primers D74949 and D74950 were used for HR1 and D74951 and D74952 were used for HR2. For insertion adjacent to *mntH* primers D75746 and D75747 were used for HR1 and D75748 and D75749 were used for HR2. Finally, the MalI binding site array was cloned on an EcoRI-NheI fragment from pUCMal20 and ligated into each plasmid containing HR1 and HR2, digested EcoRI-NheI. The resulting plasmids were pLR19 for insertion adjacent to *araBAD*, pLR17 for insertion adjacent to *araFGH* and pSB5 for insertion adjacent to *mntH*. These plasmids were used as substrates in recombineering experiments using MG1655 cells to generate strains LR17, LR38 and SXB3 respectively.
