# Supplementary material for: Development of a new fluorescent reporter:operator system: location of AraC regulated genes in Escherichia coli K-12
Source: BMC Microbiol. 2017 Aug 3;17:170. doi: 10.1186/s12866-017-1079-2 (PMC5543585; doi:10.1186/s12866-017-1079-2)

**MalI:mCherry expressed from plasmid pLER108 in MG1655**. Cells were grown in minimal media supplemented with 0.3% fructose at 23°C until they reached an OD_650_ of approximately 0.1. Hoechst 33258 was used to stain the nucleoid.

mCherry

Hoechst 33258

Brightfield

Merged


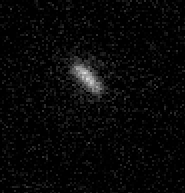

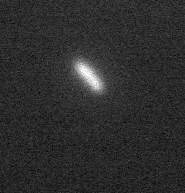

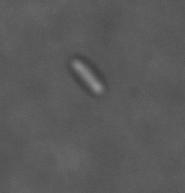

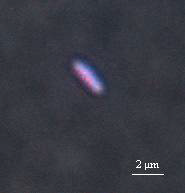

Supplement: Supplementary file 5 — MalI:mCherry expressed from plasmid pLER108 in MG1655. (DOCX 325 kb) [file 12866_2017_1079_MOESM5_ESM.docx]
